# The importance of sexuality, changes in erectile functioning and its association with self-esteem in men with localized prostate cancer: data from an observational study

**DOI:** 10.1186/s12894-019-0436-x

**Published:** 2019-01-21

**Authors:** Caren Hilger, Martin Schostak, Stephan Neubauer, Ahmed Magheli, Thomas Fydrich, Silke Burkert, Friederike Kendel

**Affiliations:** 1 Institute of Medical Psychology, Charité Universitätsmedizin Berlin, D-10117 Berlin, Germany; 2 Department of Urology and Pediatric Urology, Otto von Guericke University Magdeburg, Magdeburg, Germany; 3 Department of Urology, Klinik am Ring Cologne, Cologne, Germany; 4 Department of Urology, Vivantes Klinikum am Urban Berlin, Berlin, Germany; 5 Institute of Psychology, Humboldt-Universität zu Berlin, Berlin, Germany; 6 Department of Psychology, University of Applied Sciences Europe, Berlin, Germany

**Keywords:** Active surveillance, Erectile functioning, Importance of sexuality, Localized prostate cancer, Radical prostatectomy, Self-esteem

## Abstract

**Background:**

Treatment for localized prostate cancer (PCa) can cause long-term changes in erectile functioning. However, data on the importance of sexuality and possible consequences of altered erectile functioning on self-esteem in men with localized PCa are lacking.

**Methods:**

Self-report questionnaires were completed by 292 men with PCa, initially managed with active surveillance (AS) or radical prostatectomy (RP). Independent t-tests were conducted to evaluate group differences. A sequential multiple regression model was fitted to analyze the associations between the importance of sexuality, changes in erectile functioning and impairment of self-esteem. Interaction effects were tested using simple slope analyses.

**Results:**

Participants were 70 ± 7.2 years old and 66.5% rated sex as being “rather/very important”. The two groups differed markedly in changes in erectile functioning, importance of sexuality and impairment of self-esteem (*p* < .001), with higher values in RP patients. Regression analysis showed that after adjustment for control variables and importance of sexuality, changes in erectile functioning were still associated with impairment of self-esteem (*B* = .668, *SE* = .069, *p* < .001). The interaction of changes in erectile functioning and importance of sexuality reached significance (*B* = .318, *SE* = .062, *p* < .001).

**Conclusions:**

RP patients report more changes in erectile functioning than AS patients. Moreover, in men with localized PCa, erectile functioning and self-esteem are closely related. Sexuality seems to be important for the majority of these men. Physicians should address the possibility of erectile dysfunction and its potential effects on psychological well-being before the treatment decision.

## Background

Men who are diagnosed with localized prostate cancer (PCa) have to choose among treatment options that differ considerably in their invasiveness, [1] ranging from active surveillance (AS) to radical prostatectomy (RP). In AS, treatment is delayed until a predefined histological or biochemical disease progression occurs, or until the patient choses a different treatment strategy due to other reasons, for example, cancer-related anxiety. [2] One of the most important reasons that men opt for AS is the possible impairment of sexual function as a side effect of invasive treatment. [3] Negative effects of RP on sexual function compared to AS have been summarized in systematic reviews [4, 5] and were confirmed by the latest results from the randomized controlled trial ProtecT that compared RP to AS directly. [6] Recent research has also highlighted a close association of PCa treatment and psychosexual outcomes, such as sexual bother [7] and an impaired sense of masculinity. [8] However, most studies dealing with sexuality in PCa patients still focus on the physiological aspects of sexuality despite the fact that an impairment of erectile function has far-reaching consequences on men’s quality of life. [9, 10] For most men, sexuality is a vital aspect of their male identity and an impairment of erectile functioning may also affect their self-esteem (i.e. men’s appraisal of their individual abilities combined with a sense of self-worth [11]). [12–14]

Prostate cancer is typically a disease of older men. [16] Therefore, one may assume that an impairment of erectile functioning is negligible in most PCa patients. Eventually, at the time of diagnosis, men are an average of about 70 years old. [17] However, to draw the conclusion that sexuality plays a subordinate role in the process of decision-making could mean to adapt the stereotype of sex unjustifiably being “unseemly, even unnatural in the old.” [15] Indeed, sexual activity decreases with increasing age. Nevertheless, most older men still seem to experience sexual desire [15, 18] and also report being sexually active. [9, 19]

Although the relationship between erectile dysfunction and self-esteem has been subject to previous studies [13, 20], no study, to the best of our knowledge, has examined this association in men with localized PCa undergoing different treatment options. Additionally, data on the importance of sexuality in this population are lacking. The diagnosis of PCa might represent a critical life-event for some men. One could therefore assume that the diagnosis entails a change in men’s priorities: PCa and its treatment may become the main focus whereas other aspects of quality of life, such as sexuality, may become of diminishing relevance. In fact, studies with colorectal cancer patients showed that the importance of sexuality decreased due to the cancer experience [21]. It remains unclear, however, whether this also applies to men with localized PCa. Our study therefore seeks to find out how important sexuality actually is for men with localized PCa. In addition, PCa treatment and RP in particular can result in an abrupt change of erectile functioning (in contrast to a normative, more gradual worsening of erectile functioning with increasing age). This does not allow for anticipating non-normative changes in erectile functioning for men with localized PCa after invasive treatment, which could also have an impact on self-esteem.

This study seeks to close a twofold gap: On the one hand, we wanted to assess the consequences of erectile dysfunction on PCa patients’ self-esteem and, on the other hand, we wanted to shed light on the importance of sexuality in this population of older men with PCa. More specifically, we aimed at (1) comparing perceived changes in erectile functioning, self-esteem, and the importance of sexuality in men having opted for either RP or AS, and (2) assessing how changes in erectile functioning for the worse affect PCa patients’ self-esteem, considering the importance of sexuality.

## Methods

### Study design, procedure and participants

The study was nested within the prospective, multicenter, observational HAROW study, which included patients newly diagnosed with localized PCa between July 2008 and July 2013. [22] The present study was conducted in 2014: 378 men who had chosen RP as their primary treatment were matched based on the time since treatment decision to 378 men who had opted for AS. Men who opted for radiation therapy were excluded from this study as it aimed at comparing the two extremes of possible treatment strategies (AS = minimally invasive vs. RP = maximally invasive). All men contacted had agreed to take part in future studies. Men in the high risk category, as defined by D’Amico and colleagues, [23] were excluded from the study to improve the balance of the two treatment groups (see Kendel and colleagues for more details [24]). Those participants who gave written consent to the follow-up were mailed a self-report questionnaire. The average time from treatment decision to follow-up was 42 ± 16.6 months (range: 1–6 years).

The final sample comprised 292 men (150 RP patients, 142 AS patients). All participants had given informed consent prior to the study. IRB approval was obtained from the Charité – Universitätsmedizin Berlin (EA 1/242/13).

### Materials and main outcome measures

Baseline clinical parameters were abstracted from case report forms. Changes in erectile functioning (*“Did your erectile function change due to your prostate cancer or its treatment?”*) and impairment of self-esteem due to decreased erectile functioning (*“If your erectile function has decreased, did this impair your self-esteem?”*) were measured using items adapted from Johannson and colleagues. [25] The item measuring subjective importance of sexuality (*“How important is sexuality for you?”*) was adapted from van den Bergh and colleagues. [26] A five-point Likert scale was used for the item on impairment of self-esteem (range 0–4 with 0 indicating no changes in erectile functioning and therefore no impairment of self-esteem, 1 indicating changes in erectile functioning but no impairment of self-esteem due to these changes and 2–4 indicating changes in erectile functioning with lower to high impairment of self-esteem). For all other items, a four-point Likert scale (range 1–4) was used, with higher levels indicating higher expressions of the specific characteristic.

Previous studies have demonstrated that erectile dysfunction is not necessarily associated with decreased sexual satisfaction. [9] Therefore, satisfaction with sexual life was assessed as a control variable. The item (*“Overall, how satisfied are you with your current sex-life?”*) was adapted from van den Bergh and colleagues. [26] A five-point Likert scale was used for the item on satisfaction with sexual life (range 1–5, higher levels indicating higher expressions of satisfaction).

### Statistical analysis

The RP and AS patients were compared regarding relevant sociodemographic variables and cancer characteristics. Chi^2^-tests were run for categorical variables and independent t-tests were conducted for continuous variables to compare (a) changes in erectile functioning, (b) impairment of self-esteem (c) the importance of sexuality and (d) satisfaction with sexual life between the two groups. Cohen’s *d* as a measure of effect size was calculated for group comparisons. Bivariate correlations between variables were calculated. A sequential multiple regression analysis was conducted to evaluate the relationship between sociodemographic characteristics, clinical variables, the importance of sexuality and changes in erectile functioning with the criterion self-esteem. Covariates were entered first, followed by the importance of sexuality, changes in erectile functioning and finally the interaction term. Within all regression models, collinearity diagnostics indicated no multicollinearity among the independent variables. For moderation analyses, [27] predictor variables and the constituents of the respective interaction terms were centered around their grand means. In moderation models, covariates were entered first, followed by the importance of sexuality as moderator and changes in erectile functioning as predictor, and finally by the interaction term. To display and test the interaction effects, simple slopes were tested. [28] Low and high values of the continuous moderators were generated by adding or subtracting one standard deviation from the centered mean of the respective moderator. Moderation analyses were conducted using the PROCESS macro for SPSS (model 1). [29] An alpha level of *p* < 0.05 was considered significant for all analyses.

## Results

Of the 292 men with PCa included in this study, 150 (51.4%) had chosen RP as the initial treatment and 142 (48.6%) were still under AS. Table 1 presents the sociodemographic data, clinical characteristics and descriptive statistics based on the two different treatment groups. Table 2 displays correlations between the variables under study. Fewer men after RP (69%) were classified as low-risk compared with men under AS (88%). The two groups differed in age, marital status, occupation, and initial cancer risk classification (Table 1). The RP patients reported significantly more changes in erectile functioning (*t* (246.8) = − 10.11, *p* < .001; *d* = 1.2), and greater impairment of self-esteem (*t* (269) = − 4.19, *p* < .001, *d* = .51) than men under AS. On average, men under AS considered sexuality as being less important compared to the RP patients (*t* (278.4) = − 3.54, *p* < .001; *d* = .42). Across both groups, 66.5% of men rated sexuality as “rather or very important” (59% of the AS patients, 73.5% of the RP patients). The AS patients reported being more satisfied with their sexual life compared to RP patients (*t* (277.4) = 3.17, *p* = .002, *d* = .38). The correlation between satisfaction with sexual life and changes in erectile functioning was *r* = −.42 (*p* < .001, Table 2).

**Table 1:** Sample characteristics and sexuality

	Total (*n* = 292)	RP (*n* = 150)	AS (*n* = 142)	*P*
Age, yrs., *M (SD)*	70 (7.2)	67.9 (6.8)	72.2 (7.1)	< .001
Living with partner, *n* (%)	268 (92.4)	140 (94)	128 (90.8)	.031
Higher education, *n* (%)	116 (40)	61 (40.9)	54 (38.3)	.737
Still working vs. retired, *n* (%)	40 (13.8)	28 (18.8)	12 (8.5)	.04
Time since treatment decision (months), *M (SD)*	42 (16.6)	41.7 (16.3)	42.3 (17.1)	.774
Risk classification^1^				< .001
Gleason-Score (low), *n* (%)	221 (76.7)	85 (57.8)	136 (96.5)	< .001
PSA, *M (SD)*	6.2 (3.2)	6.9 (2.8)	5.5 (3.3)	< .001
Number of comorbidities, *M (SD)*	1.8 (1.5)	1.8 (1.5)	1.8 (1.5)	.713
Importance of sexuality, *M (SD)*	2.8 (0.8)	2.9 (0.8)	2.6 (0.85)	< .001
Changes in erectile functioning, *M (SD)*	3 (0.99)	3.5 (0.7)	2.5 (0.99)	< .001
Impairment of self-esteem, *M (SD)*	1.8 (1.15)	2.1 (1.1)	1.5 (1.1)	< .001
Satisfaction with sexual life, *M (SD)*	2.8 (1)	2.7 (1.1)	3 (0.9)	.002

AS, active surveillance, RP, radical prostatectomy; ^1^ Risk classification based on D’Amico and colleagues [23]

**Table 2:** Correlations between study variables

Variables	2	3	4	5	6	7	8	9	10	11	12	13
1. Impairment of self-esteem	.55**	.26**	.20**	−.08	−.07	.01	.11	.04	−.04	.08	.09	−.56**
2. Changes in erectile functioning	–											
3. Importance of sexuality	.23**	–										
4. Importance* changes EF	−.12	−.14*	–									
5. Age	−.16**	−.34**	.19**	–								
6. Living with partner	.04	.13*	−.05	−.03	–							
7. Higher education	.58	.03	−.04	−.03	.10	–						
8. Still working	.09	.23**	<−.01	−.59**	.08	.06	–					
9. Time since treatment decision	−.01	−.01	.03	.21**	−.08	.06	−.13*	–				
10. Gleason-Score (low)	−.27**	−.13*	.04	.09	.01	−.01	−.03	.02	–			
11. PSA	.17**	<.01	−.04	.06	−.07	−.02	−.10	−.03	−.13*	–		
12. Number of comorbidities	.11	−.07	.01	.07	−.04	−.05	<.01	.05	−.04	−.11	–	
13. Satisfaction with sexual life	−.42**	−.09	−.20**	−.01	−.01	.01	−.01	.04	.04	−.16**	−.06	–

*p < .05; **p < .01

### Multiple regression analysis

Sequential multiple regression was used to determine whether the importance of sexuality and changes in erectile functioning predicted impairment of self-esteem (Table 3). *∆R*^*2*^ refers to the amount of variance in the criterion (*impairment of self-esteem*), which is explained by the respective variables entered into the regression model at each step. Sociodemographic variables were entered in the first step and did not explain the variance of impairment of self-esteem significantly (*p* = .08). *R*^*2*^ was significantly different from zero in the second step including *treatment group* (*∆R*^*2*^ *=* .06, *p* < .001), but not in the third to fifth step including *PSA* and *Gleason-Score* (*∆R*^*2*^ = .002, *p* = .725), *time since treatment decision* (*∆R*^*2*^ = .004, *p* = .271) and *number of comorbidities* (*∆R*^*2*^ = .01, *p* = .1). Again, *R*^*2*^ was significantly different from zero when including *importance of sexuality* (*∆R*^*2*^ = .048, *p* < .001) and *changes in erectile functioning* (*∆R*^*2*^ = .207, *p* < .001). After the inclusion of *changes in erectile functioning*, the control variable *treatment group* was no longer significant. The interaction term of importance of sexuality*changes in erectile functioning also contributed significantly to an explanation of the variance (*∆R*^*2*^ = .062, *p* < .001).

**Table 3:** Multiple regression of impairment of self-esteem on the importance of sexuality and changes in erectile functioning: final model (*n* = 277)

Variables	*B*	*S.E.*	*β*	*∆R*^*2*^ (ad. *R*^*2*^)
Sociodemographic characteristics				
Age	.002	.011	.009	
Living with a partner	−.511	.216	−.117	
Higher education	−.05	.115	−.021	
Still working vs. retired	.137	.204	.042	.033
Group: RP vs. AS	.016	.143	.007	.06
Tumor risk category				
Gleason-Score (4–6)	.305	.141	.116	
PSA	.004	.019	.012	.002
Time since treatment decision (months)	.004	.004	.056	.004
Number of comorbidities	.039	.039	.051	.01
Importance of sexuality	.252	.074	.184	.048**
Changes in erectile functioning	.668	.069	.567	.207***
Importance of sexuality*changes in erectile functioning	.318	.062	.257	.062***
			*R*^*2*^ = .426 Adjusted *R*^*2*^ = .398 *F* = 14.93***	

*B*, unstandardized regression coefficient, *S.E.*, standard error; *β*, standardized regression coefficient, *AS*, active surveillance, *RP*, radical prostatectomy; **p < .01; ***p < .001

The final model explained 42.6% of variance (*R*^*2*^ = .426; adjusted *R*^*2*^ = .398), meaning that more than a third of the variability in impairment of self-esteem is predicted by our model. The pattern of results suggests that changes in erectile functioning predicted more than a fifth (20.7%) of the variability in impairment of self-esteem, whereas the contribution of the importance of sexuality was small (4.8%).

### Moderation analysis

**Fig. 1:**
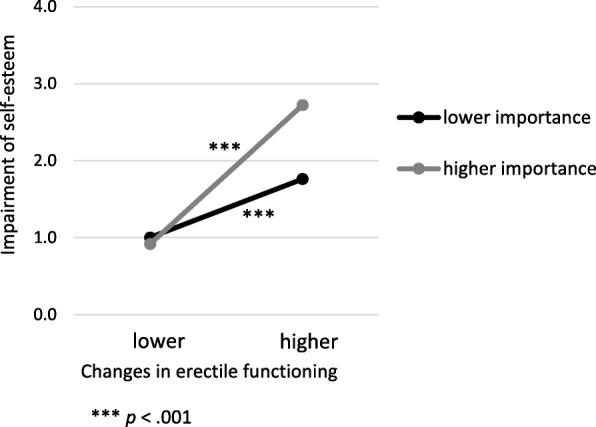
The association of changes in erectile functioning and impairment of self-esteem is stronger for men who report a higher importance of sexuality

Moderation analysis revealed that the importance of sexuality moderated effects of changes in erectile functioning on impairment of self-esteem (Table 3). Simple slope analyses showed that for both individuals with higher (*B* = .93, *t* = 10.43, *p* < .001) and lower importance of sexuality (*B* = .40, *t* = 4.78, *p* < .001) changes in sexual functioning more strongly affect their self-esteem, however this effect was weakened with lower importance of sexuality (Fig. 1). We additionally tested the conditional effect of changes in erectile functioning on impairment of self-esteem if participants reported that sexuality was “not at all” important to them. Then the effect was not significant (*B* = .11, *t* = .85, *p* = .4), i.e. if sexuality is of no importance changes in erectile functioning and self-esteem are not associated.

## Discussion

Our study aimed at evaluating the importance of sexuality and the relationship of erectile functioning and self-esteem among older men with localized PCa under different treatment strategies. We have identified three key findings: Firstly, age and, even more so, an invasive treatment for PCa are predictive of changes in erectile functioning. Secondly, even after adjustment for socio-demographic and clinical variables, changes in erectile functioning prove to be a strong predictor of self-esteem. Thirdly, the existing relationship between erectile functioning and self-esteem seems to be stronger in those men, for whom sexuality is more important.

Our finding that RP is associated with poorer erectile functioning is supported by findings of RCTs that provide evidence for the invasive procedure being a causal factor. [6, 30] In addition, the results of our study show that men after AS consider sexuality as being less important compared to men after RP. This finding may appear counterintuitive at first sight as one could assume that AS is predominantly chosen by those men who value sexuality as more important. However, due to the cross-sectional design of our study we cannot identify the time at which sexuality became more important for RP patients. We suggest that the higher importance of sexuality for men after RP manifested after surgery: erectile dysfunction is a common side-effect of RP and men and their partners have to cope with this problem in the long-term. In this process, sexuality may become a present, yet sensitive topic in men’s everyday-life, which vice versa might change men’s attitude towards sexuality.

### Erectile functioning is a strong predictor for self-esteem

Erectile functioning explains more than a fifth of the variance in self-esteem across both groups, after adjusting for sociodemographic variables, group assignment and the importance of sexuality. We challenged the common stereotype portraying older people as “asexual” by integrating the importance of sexuality in our study. [18] It is often assumed that older people may no longer consider sexuality an important aspect of their life and would, therefore, see a worsening of their sexual functioning as irrelevant. Our results contradict this stereotype: two thirds of the men, who were on average 70 years of age, rated sexuality as being “rather or very important”. However, even after adjusting for the importance of sexuality, there was a stable association between worsened sexual functioning and impaired self-esteem. This means that even men who consider sexuality as less important could experience an impairment of their self-esteem due to changes in erectile functioning. This finding corresponds with the results from a qualitative study by Gannon and colleagues, who supposed that the ability to perform penetrative sex may be essential for male identity, independent of a man’s actual sexual activity. [31] Only in the small group of men (7% of our sample), who reported that sexuality was of *no* importance to them, there was no association between erectile functioning and self-esteem.

### Changes in erectile functioning may have different consequences for men with localized PCa

Our results show a strong association of erectile functioning and self-esteem for men with localized PCa. Moreover, altered erectile functioning and satisfaction with sexual life are negatively correlated in our sample. In a study with men from the general population Braun and colleagues demonstrated that only 14% of men aged 60 to 69 years report having ED and being dissatisfied with their sex life. [9] In our study, however, almost 40% of men who report at least some changes in erectile functioning state that they are “dissatisfied or very dissatisfied” with their sex life. Thus, the consequences of erectile dysfunction might be different for men with localized PCa compared to men without PCa. After the diagnosis, men might address actual or anticipated erectile dysfunction as a possible consequence of invasive PCa treatment. The intensive preoccupation with implications of erectile dysfunction may thus strengthen the association of erectile functioning and self-esteem, whereas for men without PCa a decrease in erectile functioning may be a simple expression of a normal aging process.

### Strengths and limitations

The strengths of this study are a high external validity by using a non-interventional, multicenter design, the length of the follow-up and the sample size which represents one of the largest samples for the comparison of RP and AS. Additionally, our study focuses on the psychosocial aspects of sexuality. While most studies addressing sexuality in men with localized PCa focus more on physiological aspects, we aimed to investigate the psychological aspects of an altered sexuality. We selected our items measuring men’s sexual life accordingly. However, the study is subject to some limitations. (1) The cross-sectional and observational design does not allow for causal interpretations. For example, it remains speculative whether a change in erectile functioning for the worse leads to an impairment of self-esteem, or whether there are additional confounding variables. To deal with possible selection biases due to the non-randomization, we controlled for several sociodemographic and clinical variables, which did not explain a significant amount of variance. As opposed to a randomized study, strengths of our observational design are a high external validity and an exceptional participant rate. (2) Our data are based on the participants’ self-reports of erectile functioning and we did not validate the latter by objective measures (e.g. medical records). However, the focus of the study was on men’s subjective experience, which, particularly regarding sexuality, seems more important than objective parameters. [32] (3) Information on the psychometric properties of measurement of sexuality used in our study is still lacking. (4) Impairment of self-esteem was assessed in the context of erectile functioning. As 90% of our sample report having experienced at least some degree of changes in erectile functioning it seems reasonable to measure self-esteem in this way. However, the amount of variance in self-esteem explained by erectile functioning in our findings may be artificially inflated as a consequence. Thus, independent measures of (impaired) self-esteem should also be taken into account in future research. (5) We did not assess whether men with impaired sexual functioning made use of erection restoration treatments, which could have had an impact on our results. Therefore, this aspect should be included and analysed in future studies. Moreover, we are well aware that a differentiation between homosexual and heterosexual men’s sexuality is desirable. The importance of sexuality and erectile functioning particularly when predicting self-esteem could differ among these subgroups. Therefore, our findings may not generalize regarding homosexual men. Finally, our sample comprises a cohort that was operated in the years 2008 to 2013. Since then, great progress has been made in the field of nerve-sparing PCa surgery. Hence, compared to the present situation, the differences between AS and RP patients in this study may be more pronounced, and emphasis should be given to the relationship between erectile functioning, the importance of sexuality and self-esteem rather than to the absolute differences.

### Implications

Our results have some implications for future research. Firstly, questionnaires on male sexuality in PCa patients to date, focus primarily on the function rather than psychological aspects of sexuality. An effort should be made to develop questionnaires that comply with a broader definition of sexuality as a holistic construct [15] and meet the particularities of older men or PCa patients. Widely accepted and validated questionnaires would also facilitate a comparison of results. Secondly, longitudinal studies in this field are desirable to gain a deeper insight into the interplay of age, sexuality, self-esteem and other psychosocial variables in men with PCa over time. As erectile functioning is only one facet of the sexual response cycle that can be affected by invasive PCa treatment more items assessing sexual functioning should be included. Similarly, self-esteem is only one example for the connection between sexual functioning and psychological well-being. Future research should take this into account.

Other implications refer to clinical practice. Previous studies have shown that psychosocial interventions providing information or emotional support can improve psychological outcomes in men with localized PCa. [33] However, men with localized PCa stated not being in need of psychosocial interventions in a qualitative study. [34] Thus, despite of being potentially beneficial for men with localized PCa the preliminary setting of psychosocial interventions may not meet men’s needs. We therefore suggest low-threshold services to counsel men on PCa treatment, possible physiological side-effects and associated psychological consequences. This could be put into practice using online tools or by extending the role of the urologist: For men with localized PCa, the urologist is usually the first and probably the most important specialist contact person. With urologists not only giving medical but also psychological advice PCa patients would receive comprehensive consulting without having to make additional efforts. A specific clinical implication of our study concerning medical practice is to structure the doctor-patient conversation about sexuality. Many men still have difficulties discussing this topic with their physician. However, they feel their physician should address it on his or her own volition. The discussion about adverse effects of an invasive treatment should comprise the psychological impact of an impairment of erectile functioning to reach a fully informed decision. Furthermore, the physician is crucial for rebutting stereotypes for men who experience an impairment of erectile functioning due to invasive treatment or age. The physician may discuss with the patient that the stereotype of older people being asexual, focusing mainly on the degree of erectile functioning, is based on a “too narrow definition of sexuality.” [18] Professionals could encourage their patients to maintain their sexual life and, when being sexually active, to use “what they have.” [15] Low-threshold services (e.g. online consulting tools) and interventions aiming to improve the communication of sensitive (psychological) topics should be conceptualized and tested in future research.

## Conclusions

The effect of erectile dysfunction on men’s self-esteem is a neglected topic in PCa research that deserves attention in both clinical practice and research. Changes in erectile functioning may become particularly important if they occur due to a PCa treatment. Our results emphasize the importance of sexuality for men with PCa. Therefore, medical advice on PCa treatment should encompass the strong association of erectile dysfunction and self-esteem in men with PCa. This topic should be addressed as a possible consequence of invasive treatment.

## Abbreviations

AS: Active surveillance
E.g: Exempli gratia
I.e: Id est.
PCa: Prostate cancer
RP: Radical prostatectomy
